# Engaging Patients in the Canadian Real-World Evidence for Value in Cancer Drugs (CanREValue) Initiative: Processes and Lessons Learned

**DOI:** 10.3390/curroncol29080443

**Published:** 2022-08-07

**Authors:** William K. Evans, Pam Takhar, Valerie McDonald, Martine Elias, Louise Binder, Stéphanie Michaud, Mina Tadrous, Caroline Muñoz, Kelvin K. W. Chan

**Affiliations:** 1 Department of Oncology, McMaster University, Hamilton, ON L8S 4L8, Canada; 2 Ontario Health (CCO), Toronto, ON M5G 2L3, Canada; 3 Independent Patient Representative, Toronto, ON M6G 2V3, Canada; 4 Myeloma Canada, Dorval, QC H9P 2V4, Canada; 5 Save Your Skin Foundation, Penticton, BC V2A 0B2, Canada; 6 BioCanRx, Ottawa, ON K1H 8L6, Canada; 7 Leslie Dan Faculty of Pharmacy, University of Toronto, Toronto, ON M5S 3M2, Canada; 8 Temerty Faculty of Medicine, University of Toronto, Toronto, ON M5S 1A8, Canada; 9 Canadian Centre for Applied Research in Cancer Control, Toronto, ON M5G 2L3, Canada; 10 Sunnybrook Health Sciences Centre, Toronto, ON M4N 3M3, Canada

**Keywords:** patient engagement, real-world evidence, health technology assessment, reassessment

## Abstract

The Canadian Real-world Evidence for Value in Cancer Drugs (CanREValue) Collaboration established the Engagement Working Group (WG) to ensure that all key stakeholders had an opportunity to provide input into the development and implementation of the CanREValue Real-World Evidence (RWE) Framework. Two consultations were held in 2021 to solicit patient perspectives on key policy and data access issues identified in the interim policy and data WG reports. Over 30 individuals, representing patients, caregivers, advocacy leaders, and individuals engaged in patient research were invited to participate. The consultations provided important feedback and valuable lessons in patient engagement. Patient leaders actively shaped the process and content of the consultation. Breakout groups facilitated by patient advocacy leaders gave the opportunity for open and thoughtful contributions from all participants. Important recommendations were made: the RWE framework should not impede access to new drugs; it should be used to support conditional approvals; patient relevant endpoints should be captured in provincial datasets; access to data to conduct RWE should be improved; and privacy issues must be considered. The manuscript documents the CanREValue experience of engaging patients in a consultative process and the useful contributions that can be achieved when the processes to engage are guided by patients themselves.

## 1. Introduction

The Canadian Real-world Evidence for Value in Cancer Drugs (CanREValue) Collaboration was established in 2017 with a Health Systems Improvement grant from the Canadian Institutes of Health Research. The goal of this research project is to develop a framework to generate and use real-world evidence (RWE) for the evaluation of the effectiveness and safety of cancer drugs following their initial health technology assessment (HTA). The need for such a process is driven by the fact that many manufacturers’ drug submissions are based on immature survival data and insufficient information about adverse events and long-term side effects. In addition, the molecular definition of cancer subtypes has resulted in the identification of some very small patient populations that preclude generating data on clinical benefit from robust randomized controlled phase III trials. With the ever-increasing number of innovative, effective but expensive anticancer drugs, payers increasingly want solid evidence that the new agents provide value for money.

**Figure 1:**
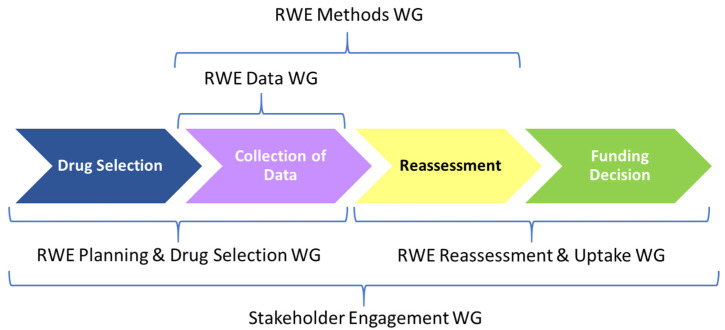
The Canadian Real-world Evidence Value of Cancer Drugs (CanREValue) project real-world evidence (RWE) working groups (WGs) and their roles within the development of the framework.

The work of the CanREValue collaboration is being undertaken by five working groups (WG), each focused on developing specific processes for the generation and use of RWE in decision-making about cancer drug funding (Figure 1). The five WGs are: (1) RWE Planning and Drug Selection; (2) RWE Data; (3) RWE Methods; (4) RWE Reassessment and Uptake and (5) Engagement. Each WG is comprised of members with expertise in drug approval, drug pricing, and HTA processes. Furthermore, there are public payer, clinician, and patient representatives on each WG.

**Figure 2:**
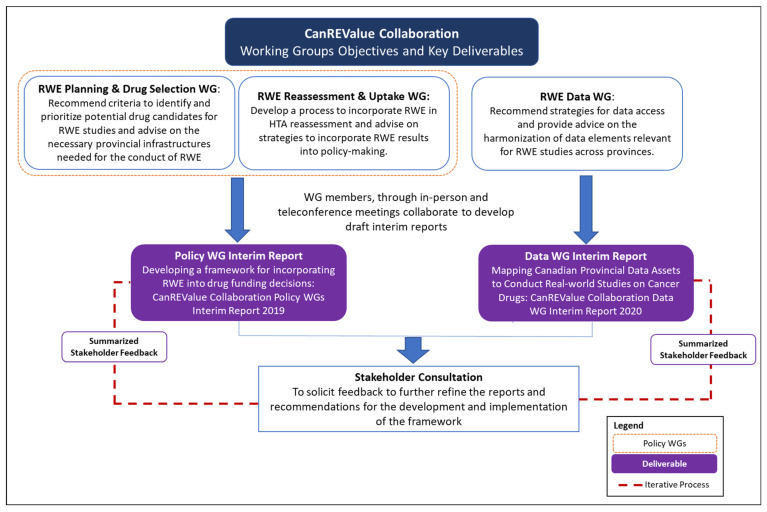
The CanREValue Policy and Data Working Group Interim Report Process.

As is shown schematically in Figure 2, the Planning and Drug Selection WG and the Reassessment and Uptake WG produced an interim policy report through a series of in-person and teleconference meetings [1]. This interim policy report describes the factors to be considered in determining the feasibility of potential RWE projects and conducting a reassessment review, as well as preliminary models for the identification and selection of RWE projects and the reassessment process. Similarly, the Data WG produced an interim report based on its review of publicly available databases and the data elements within these databases that could potentially be used to undertake RWE studies [2]. The Data WG also identified where enhancements to data collection would need to be made to support such studies.

To solicit feedback, the core team posted the WG reports on the Canadian Centre for Applied Research and Cancer Control (ARCC)/CanREValue website (https://cc-arcc.ca/canrevalue/) and reached out to the CanREValue mailing list, requesting that respondents review the reports and answer three survey questions [1,3]: What barriers and opportunities do you see to the implementation of the proposed framework?; What benefits/opportunities are there for your organization if the proposed framework for the reassessment of funded drugs is implemented? What role would you or other stakeholders like in the development and implementation of the framework? Feedback on these interim reports was received from academics, individual industry representatives, industry-related organizations, and patient representatives [4,5]. The feedback was provided to the WGs through their Chairs and the report documents were modified and updated based on the consensus view of the WG members.

When patient leaders approached the CanREValue leadership seeking opportunities to participate, the Engagement WG set up a process to actively solicit patient input through two virtual consultations with patient representatives.

## 2. Approach

### 2.1. Stakeholder Engagement Process

The role of the Engagement WG was to ensure that all parties interested in or potentially impacted by RWE were made aware of the CanREValue project and were given ample opportunity to provide input into the development of the framework. The format for consultations was to review the progress on the development of the RWE framework, to present each issue that had been received in feedback to the interim reports and to solicit further comments on the feedback and the CanREValue response [1,2,4,5].

Four prominent patient advocates (LB, ME, SM, CS) and a former patient representative on the expert review committee for oncology drugs at CADTH (VM) (Louise Binder, Save Your Skin Foundation; Martine Elias, Myeloma Canada; Valerie McDonald, former patient representative on the CADTH HTA Committee for Oncology Drugs; Stéphanie Michaud, BioCanRx and Christina Sit, Lung Cancer Canada) were invited to serve as an organizing committee for the patient consultation. The number of participants had to be limited because of ongoing COVID restrictions and in order to be workable. Their first task was to identify individuals in their networks who they felt would be interested in reviewing and discussing the interim WG reports [1,2,4,5]. They identified 32 individuals who were either patients, caregivers, advocacy leaders, or individuals engaged in research of patient-related issues. These individuals were contacted by email and invited to participate in the consultation process. The email provided a brief explanation of the CanREValue project and the purpose of the consultation. Those who accepted the invitation were then polled on the most suitable date.

### 2.2. Identification of Topics of Greatest Interest to Patients

The patient organizing committee reviewed the slide deck that had been used in previous consultations and revised it to focus on those issues which were of greatest importance to patients (Table 1). The topics chosen were the definition of RWE, the triggers for undertaking RWE studies and the recommendation that delisting a drug could be one potential outcome from a RWE assessment [1,4]. In addition, the organizing committee wanted to discuss how the CanREValue framework could be used when the HTA resulted in a conditional recommendation. Feedback on the interim Data WG report was sought, especially regarding concerns about data access and “missing” data elements in the publicly accessible provincial databases [2]. 

Prior to the consultation session, relevant materials (e.g., a list of key issues, slide deck presentation, WG reports) were shared. Participants were asked to review the Policy and Data WG reports, as well as a manuscript that described the CanREValue initiative [1,2,4,5,6]. In addition, to facilitate the virtual meeting, pictures and short biographies of the participants were distributed prior to the meeting to help those attending know who else was present and to recognize them in the virtual meeting.

### 2.3. Format and Feedback: The First Consultative Session

The first Patient Engagement Consultation took place on 24 August 2021 and was scheduled for 1.5 h. After a welcome from the CanREValue leadership, the roles of the various WGs were reviewed, and the component parts of the draft framework were presented.

The issues raised in the feedback process were then presented sequentially and the CanREValue response to each issue was described. For each of these issues, the patient perspective was sought. To prompt discussion, participants were asked:

1. Do you have any further thoughts on the feedback received?
2. Has our response addressed the concerns raised?
3. How might we improve on the response?
4. Are there other issues related to “policy” that are important from a patient perspective that have not been raised?

### 2.4. Format and Feedback: The Second Consultative Session

The second Patient Engagement Consultation was scheduled for 2 h on 21 October 2021. Introductions, and review of the CanREValue framework were kept brief to provide time for discussion. Based on the feedback received following the first consultation session and to maximize the opportunity for all participants to contribute, two simultaneous breakout groups were held. Each was led and facilitated by a member of the organizing committee, supported by a member of the CanREValue leadership and a reporter who reported back to the main group.

Following the second session, the core team compiled a summary document based on what was discussed during the consultations plus comments received by email following each session. Once finalized, the summary document was shared amongst the participants to ensure accuracy and completeness. The summary document was also shared for comment with the patient representatives who had expressed interest but were unable to attend.

## 3. Results

Over 20 individuals from across Canada representing patients, caregivers, advocacy leaders, and individuals engaged in patient research participated in the consultation sessions. The majority of the participants were female and had firsthand experience of living with cancer or a rare disease. All participants had experience in patient advocacy work (e.g., involved in non-profit organizations, served as patient representatives on HTA organizations, etc.). In Table 2 below the key feedback received is summarized.

### 3.1. Findings: The First Consultative Session

#### 3.1.1. Definition of RWE

The response to the feedback on the definition of RWE was mixed. Some participants felt that the definition used in the WG documents was “narrow”, while others viewed it as appropriate. However, when it was explained that the CanREValue definition aligns with that of the FDA and other international organizations, the participants accepted the definition being proposed by the WG.

#### 3.1.2. “Triggers” for Undertaking RWE

The Planning and Drug Selection WG received feedback that the “triggers” or signals that might indicate the need to undertake RWE studies were quite broad. In particular, there was concern that the value for money trigger could be applied to almost every HTA recommendation. The WG responded that the list of potential triggers was meant to be comprehensive in order to address the full range of uncertainties that might be encountered. It was acknowledged that the triggers would have to be prioritized based on the needs of each RWE project and that prioritization of the triggers would be one of the next steps in its work.

#### 3.1.3. Barriers to Effective Decision-Making

Patient representatives identified several barriers to effective decision-making, the most important of which is immature survival data. Participants noted that more mature data could be obtained simply by delaying decision-making to allow for trial data to mature but this would create access delays. To address the gaps in data, however, patient representatives suggested that some data, such as toxicity data and quality of life data, might be acquired through compassionate release programs. Consent and privacy issues would have to be addressed. It was acknowledged that there would need to be data priorities identified to minimize the burden of data collection on both patients and physicians and to only capture data needed to address the specific RWE question. Because of the variability in the data collected in provincial databases, there was a strong consensus that a national initiative needed to be undertaken to develop standardized data definitions, to identify essential new data elements (e.g., performance status, quality of life data) and to improve the capacity of provinces to collect these data.

#### 3.1.4. Other Issues Raised

The patient representatives expressed concern that health system sustainability and cost containment seemed to be the key drivers for the RWE framework rather than concerns about access to new and effective agents for patients. In support of this concern, patients pointed to a reference in the interim Policy WG paper that appeared to give precedence to payers in the decision-making priorities. The interim report stated that efforts would “focus on projects that are of relevance to stakeholders and to payers, in particular” [1] (p. 14). The patient representatives acknowledged the reality of limited drug funding budgets and the potential for RWE to help cancer agencies to better allocate limited funds to maximize health benefits to patients. The CanREValue leadership felt that RWE had potential to improve timely access to treatments, but the patient perspective was needed to define value, as patients value therapeutic gain and quality of life. Payers, on the other hand, may define value primarily in monetary terms. Again, participants pointed to wording in the interim Policy WG report (p. 12) that failed to mention outcomes of importance to patients, such as progression-free survival, quality of life, and patient reported outcomes or experiences [1].

At the conclusion of the first consultation, participants were encouraged to reflect on the issues discussed and email any additional thoughts/feedback they had. There was strong support for a second consultation. Email feedback indicated that some patient representatives felt they had not been given the opportunity to contribute. The organizing committee discussed how best to address the concerns raised by participants. It was strongly felt that one large virtual group made it difficult for some representatives to have the opportunity to speak up and that the next session should have breakout groups with a smaller number of participants so that all could participate.

### 3.2. Findings: The Second Consultative Session

Each breakout group considered three issues: whether the “delist” recommendation threatened access to medications; whether the framework should apply only to currently reimbursed drugs; and what strategies might be used to address the missing data elements important for RWE studies. Participants were prompted with the discussion questions used in the first consultation session and an additional prompt: Given that the review of the publicly accessible administrative datasets identified missing data elements and access issues, what actions could/should patient groups undertake to improve the availability of data for RWE?

#### 3.2.1. “Delist” Recommendation

The topic of delisting previously approved drugs generated the greatest amount of discussion. Participants acknowledged the need to consider delisting as a recommendation and that removing the option undermined the sustainability of the healthcare system. However, discussion revealed that new, more effective treatment regimens typically displace older, less effective approaches, essentially delisting them. Nonetheless, participants felt it was important that the potential for some drugs to be delisted be discussed and widely communicated so that there would be buy-in by all stakeholders, including patients. The CanREValue leadership acknowledged that the purpose of RWE is not to delist treatments but rather to address uncertainties about the value of new interventions, which could facilitate access to new treatments. There was discussion about the terminology itself and whether the term “delist” conveyed the notion that a treatment option was being taken away from patients. Rather than changing terminology, it was concluded that more effort was needed to explain how the RWE process worked and to clarify the distinction between delisting a drug that has already been through the approval processes versus not approving a new drug.

The majority of participants supported the development of a “grandfather” clause to complement the delist recommendation to allow for continued access to drugs for patients who were benefiting from them. Delist recommendations need to indicate the reasons for delisting. For example, if a drug was delisted due to safety concerns, patients who were tolerating the drug or managing its toxicities should be able to continue to access it. This decision to continue receiving a delisted drug should be made by the patient and his/her physician.

Finally, participants underscored the need to create a cohesive ecosystem in which RWE could be utilized to facilitate early access to innovations while delisting less-effective products. Participants emphasized that the framework should not encourage industry to present immature data in order to get their products to market early. Rather, a balance between early access and the maintenance of high-quality evidence in support of the value of new therapeutics needs to be struck.

#### 3.2.2. Conditional Funding Recommendations

The breakout groups suggested that the framework should apply not only to currently reimbursed drugs but also to new agents given a conditional funding recommendation. Participants felt that this feedback might motivate healthcare providers, patient advocates, and other groups to demand that HTA committees and the provinces increase evidence generation for new therapeutic agents. As a result of precision oncology, clinical trial numbers are often small. Collecting real world data (RWD) could expand knowledge of a drug’s effectiveness and safety. The example of chronic myelogenous leukemia was cited and how ongoing evidence generation on the use of tyrosine kinase inhibitors had improved patient management [7].

#### 3.2.3. Data Elements

Participants suggested that there needed to be a compilation of the data elements that are needed for RWE studies but are not currently captured in provincial databases [2]. It was noted that provinces do not all capture the same data, which limits research, and that access to publicly administered databases is often difficult. Patients were particularly concerned by the failure of the administrative datasets to capture data elements that patients value and the limited data on First Nations populations, populations living in remote parts of Canada and other under-served patient groups. The participants stressed the importance of engaging with patients to understand and prioritize what they consider to be valuable endpoints and outcomes, including quality of life, disease-free survival, and progression-free survival. These endpoints need to be captured in the generation of RWE. There was discussion of the need for a national approach to data access, data gathering, and data sharing. The potential to use artificial intelligence in data gathering was suggested and building this capability might be supported through public–private partnerships.

#### 3.2.4. Other Issues Raised

One issue that was of particular importance to the participants was the under-representation of patients in the CanREValue WGs. Specifically, there was concern that having only a single patient representative on a committee is inadequate because of the power dynamics. Participants expressed the view that having only one or two patient representatives on committees often results in patients feeling uncomfortable about speaking up. Therefore, the recommendation was made that patient representation on the CanREValue committees be expanded. The CanREValue leadership acknowledged the issue and noted that the individual WGs are quite large and reflect a very diverse stakeholder perspective. Further, the Engagement WG was specifically established to ensure that there was adequate engagement of all stakeholder groups, including patients. Finally, participants questioned how decisions about the RWE project would be communicated to patient groups and whether there will be future opportunities to provide input into potential RWE studies. CanREValue is committed to engaging with and seeking input from patient groups both through the posting of WG reports online and soliciting feedback and further consultative sessions as the framework is developed and refined.

## 4. Discussion

Engagement with patient representatives through two virtual consultations proved to be highly informative and resulted in a better understanding of the patient perspective on key policy and data access issues relating to RWE. The importance and benefits of public engagement, more specifically, patient and caregiver engagement, within the health policy and health technology assessment landscape in Canada and internationally is widely recognized and gaining momentum [8,9,10]. Engaging patient representatives helps to identify unmet needs, improves health outcomes, promotes collaborative decision-making and implementation, and ensures that the patient perspective informs the health technology assessment process [9,10,11].

The consultations proved to be instructive from a number of perspectives. Firstly, we identified that the consultative process had to provide an environment that allowed an open, honest communication. Successful patient engagement hinges on providing adequate time for discussion, a supportive environment where participants have the opportunity to be heard, and a process for monitoring satisfaction among patient participants [8]. Following the initial consultation, patient representatives provided direct feedback to the CanREValue team allowing the patient advocacy leaders and the CanREValue team time to debrief and discuss successes and failures. The initial virtual consultation with over 20 patient representatives had inadequate time and limited opportunity for all voices to be heard. The issue of limited discussion time was addressed more effectively in the second session by reducing the time spent reviewing the CanREValue project status and providing more time for discussion in small breakout groups. Secondly, we found that the engagement of well-known Canadian patient leaders to form an organizing committee was extremely helpful in defining the topics of greatest interest for the patient representatives to discuss, in framing these issues in the presentation materials, and in facilitating the small group discussions. The organizing committee’s ability to highlight areas of interest among patients resulted in a more fulsome consultation during the second session and generated considerable valuable feedback on more effective ways to solicit patient perspectives.

Our consultations with individuals who represented patients and caregivers provided the firsthand experience of those living with cancer [8,12]. Given their unique perspective, patients and caregivers are able to share their preferences and identify gaps in important endpoints/outcomes and provide insights on what is important and of value to patients [8,12]. The CanREValue leadership heard concerns about how RWE might impede access to new drugs to expanding the vision of how the RWE framework might be used to support conditional drug approvals. There was substantial concern around the potential for previously approved drugs to be delisted but open conversation around this topic was informative and helped to address patients’ concerns. However, there will be an ongoing need for careful communication around drug delisting as the CanREValue framework is further developed and implemented. Another important issue was the inclusion of data elements considered to be of value to patients, such as disease-free survival, progression-free survival, and quality of life, that are not currently captured in provincial health administrative databases. CanREValue stakeholders have identified a need for a national initiative to improve and expand data collection, access, and sharing to enable high-quality RWE studies. Patient advocates with an understanding of RWE studies have the knowledge, experience, and support to propel this initiative forward.

In our future work, these and the other patient perspectives documented in this manuscript will help to further refine the WG documents and ultimately provide an actionable framework. The consultation sessions reminded CanREValue members of the value of the patient voice in shaping the recommendations for the generation and use of RWE for oncology drug funding decisions in Canada.

## 5. Conclusions

The consultations to solicit patient perspectives on key policy and data access issues identified in the interim CanREValue Policy and Data WG reports provided important feedback on the RWE framework but also valuable lessons on effective patient engagement. Several important recommendations were made, including that the framework should not impede access to new drugs; the RWE framework should be used to support conditional approvals; patient relevant endpoints should be captured in provincial datasets; access to data to conduct RWE should be improved; and privacy issues must be considered. Furthermore, collaborating with well-known Canadian patient leaders was helpful in identifying patient representatives and in achieving effective and meaningful engagement. Findings from this experience will help to ensure that the development and implementation of the CanREValue RWE Framework is reflective of all key stakeholder perspectives.

## Figures and Tables

**Table 1:** Key Issues Discussed by Patient Representatives.

Key Issues
1. Definition of real-world evidence (RWE) too “narrow”
2. “Triggers” for undertaking RWE studies are quite general; value for money trigger could apply to most CADTH/INESSS recommendations
3. “Delist” recommendation should be removed; threatens access to medications
4. Framework should apply to new drug approvals and be an option for coverage with evidence development
5. Missing data elements should be identified and compiled for future action
6. Other issues raised

**Table 2:** Summary of Key Feedback.

Key Issues	Summary of Key Feedback
1. Definition of real-world evidence (RWE) too “narrow”	Some participants found the definition to be too narrow, while others felt it was appropriate. However, upon understanding that the definition aligns with that of the FDA and other international organization, participants were satisfied with the adequacy of the definition.
2. “Triggers” for undertaking RWE studies are quite general; value for money trigger could apply to most CADTH/INESSS recommendations	Participants agreed that the triggers were quite broad and expressed concern that the value for money trigger could be applied to almost every HTA recommendation. From the patient perspective, it is important to consider value in non-monetary terms; in particular, progression-free survival and quality of life are valued by patients.
3. “Delist” recommendation should be removed; threatens access to medications	Participants acknowledged the need to consider delisting as a recommendation; retaining the option to delist is important for healthcare system sustainability. The concept of delisting drugs needs to be widely discussed and communicated so that there is buy-in by all stakeholders, including patients. Alternative terminology to “delist” was discussed; however, participants felt it would be more productive to put greater effort into explaining how the RWE process works and to clarify the difference between delisting an approved drug as opposed to not approving a new drug. Most participants supported the development of a grandfather clause to complement the delist recommendation, allowing continued access to drugs for patients who were continuing to benefit from them. Important to focus on developing a cohesive ecosystem for early access and for delisting drugs. A balance needs to be struck that enables early access to potentially effective new drugs while supporting processes that remove drugs that are not providing therapeutic value. Evidence thresholds need to be retained, not lowered.
4. Framework should apply to new drug approvals and be an option for coverage with evidence development	The framework should not only apply to currently reimbursed drugs but also to new agents given conditional funding recommendations.
5. Missing data elements should be identified and compiled for future action	Participants noted that not all provinces consistently capture data nor do they capture data elements that patients value (e.g., quality of life, disease-free survival, progression-free survival); there is limited data on First Nations populations and other under-served patient groups. There is recognition of the need for a national approach to data access, data gathering and sharing. There was an acknowledgment of the need to set priorities around the amount and type of data to collect in order to avoid/reduce the burden on clinicians and patients with respect to data collection. The potential to use artificial intelligence in data gathering was suggested and building this capability might be supported through public–private partnerships.
6. Other issues raised	Participants identified barriers to effective decision-making; immature survival data is most common. Patient representation on working groups should be revised to take account of power dynamics. Concerns were raised among participants about how decisions about the RWE project would be communicated to patient groups and whether there will be future opportunities to provide input into potential RWE studies. *These questions will need to be addressed as the project matures and the WGs consider this feedback.*

## Data Availability

Not applicable.
